# Complete genome sequence of *Escherichia coli* O157:H7 phage Φ241

**DOI:** 10.1128/mra.00106-24

**Published:** 2024-07-22

**Authors:** Zhongjing Lu, Tsai-Tien Tseng

**Affiliations:** 1 Department of Molecular and Cellular Biology, College of Science and Mathematics, Kennesaw State University, Kennesaw, Georgia, USA; Montana State University, Bozeman, Montana, USA

**Keywords:** Phage Φ241, *Escherichia coli *O157:H7, complete genome sequence, biocontrol agent

## Abstract

We report the genome sequence of phage Φ241 infecting *Escherichia coli* O157:H7. Phage Φ241 was isolated from an industrial cucumber fermentation at high acidity (pH 3.7) and high salinity (5% NaCl). The phage genome consists of a 157,291 bp circular double-stranded DNA with 203 coding regions and 44.96% GC content.

## ANNOUNCEMENT

*Escherichia coli* O157:H7 is an important foodborne pathogen and a major public health concern. It can cause severe hemorrhagic colitis and life-threatening hemolytic uremic syndrome (1 – 4). The pathogen has a low infectious dose (5, 6) and high resistance to multiple drugs (7). It can survive high concentrations of NaCl (8) and low-pH environments such as gastrointestinal tracts (9) and various acidic foods (10, 11). Multistate foodborne outbreaks of *E. coli* O157:H7 infections frequently occur (12 – 18). The annual cost of illness due to *E. coli* O157:H7 infections in the United States was 405 million dollars (8) (19). To improve food safety, more effective approaches to control *E. coli* O157:H7 are much needed.

A lytic phage, Φ241, specifically infecting *E. coli* O157:H7 was isolated from an industrial cucumber fermentation at high acidity (pH 3.7) and high salinity (5% NaCl). Sample collection, phage isolation, and the biological characteristics of this phage were described previously (20). Phage Φ241 belongs to the class of Caudoviricetes. The high lytic activity, specificity, and tolerance to low pH and high salinity make Φ241 a promising biocontrol agent of *E. coli* O157:H7. To assess the genetic safety of Φ241 for its practical application, the phage genome was sequenced and is reported here.

The phage DNA was extracted through phenol:chloroform:isoamyl alcohol extraction (20). The library was constructed using the SMRTbell template prep kit 1.0. with blunt-end SMRTbell adapter. SageELF was used for size selection to a final size of ~20 Kb (Sage Science, Inc., Beverly, MA). The genome was then sequenced on the PacBio RS II platform using the P5 DNA polymerase with C3 chemistry (P5-C3) in one SMRT cell that generated 49,780 raw reads, 1,163,965,016 bases, and N50 read lengths of 26,339. The average reference coverage was 3,991.2. Genome assembly, read quality control, error correction, and adapter trimming were performed by using Hierarchical Genome Assembly Process 3 (HGAP.3) (21).

**Fig 1:**
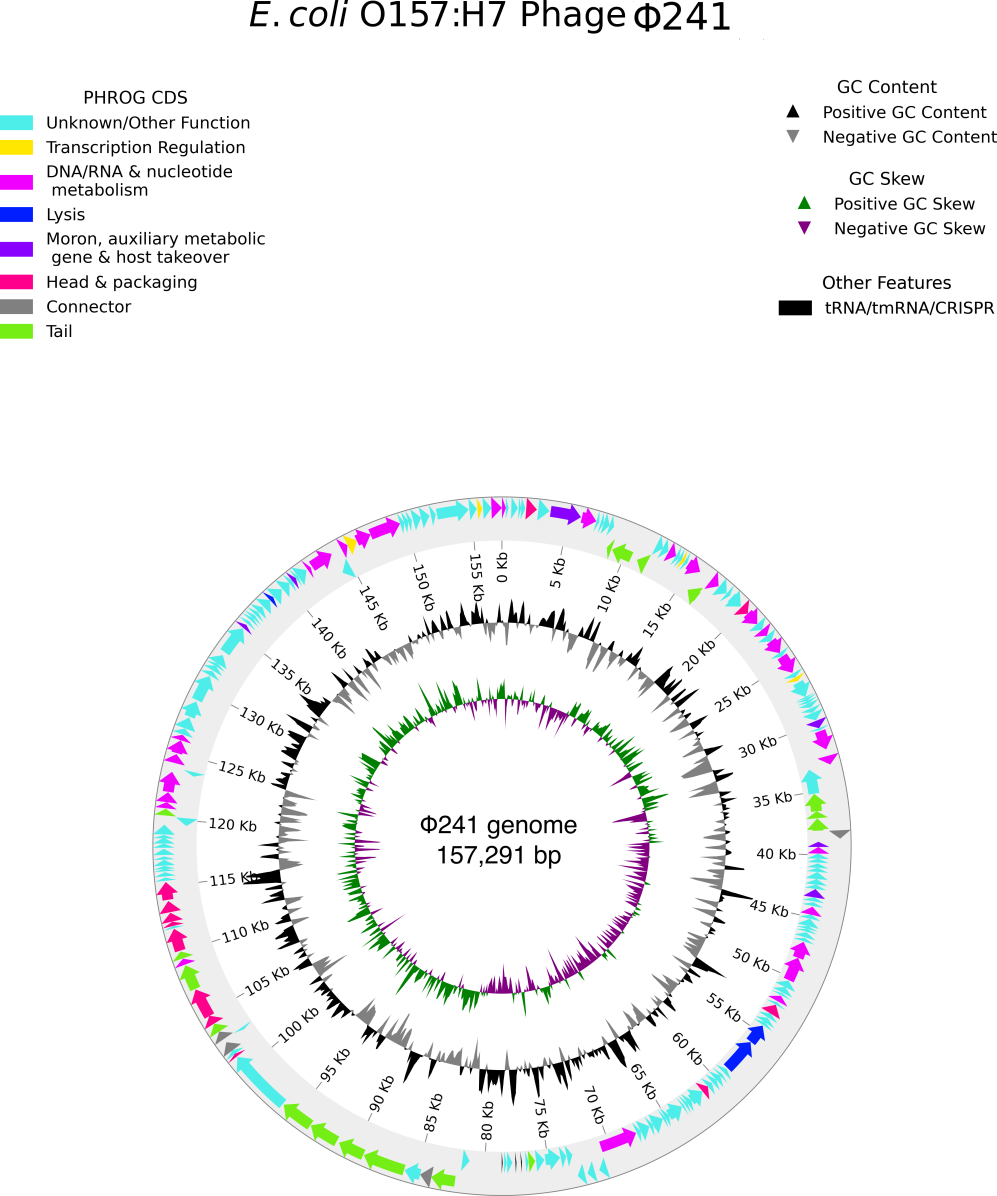
Genome map of phage Φ241. Predicted coding sequences are denoted by colored arrows with transcriptional direction and predicted function: unknown/other function (sky blue); transcription regulation (yellow); DNA/RNA and nucleotide metabolism (deep magenta); lysis (blue), moron, auxiliary metabolic gene, and host takeover (purple); head and packaging (red); connector (gray); and tail (green).

Genomic annotation was conducted using the integrated pipeline of Pharokka 1.3.2 (22). The gene function was predicted using Prodigal, as implemented in Pharokka 1.3.2 (22). Phage Φ241 has a circular double-stranded DNA genome consisting of 157,291 base pairs with a GC content of 44.96% and 203 predicted coding sequences predicted using Prodigal 2.6.3 (23). The genome map with open reading frames (Fig. 1) was generated using pharokka_plotter.py (22, 24). Eighty-nine coding sequences have a predicted function. Most of the annotated genes code for phage-related proteins such as endolysin, major head protein, head scaffolding protein, portal protein, tail proteins, connector, terminase, and host takeover proteins. Three tRNA genes were identified in the genome by using tRNAscan-SE (25). The resulting tRNAs are for carrying isoleucine, tyrosine, and methionine. The genome does not contain CRISPRs, tmRNAs, virulence factors, or antibiotic resistance genes. This suggests that Φ241 is safe to be used in foods to control *E. coli* O157:H7.

Further analysis using MegaBLAST (26) against the NCBI nucleotide database indicated that Φ241 shares 98.44% nucleotide identity with *Escherichia* phage ECML-4 (accession number NC_025446.1) and 185 hits in similar proteins. In addition, Φ241 shares 97.83% identity and 196 potential homologs with *Salmonella* phage Metapan (accession number MN066127.1).

## Data Availability

The genome sequence of phage Φ241 was deposited in GenBank and BioProject under accession numbers OR924452 and PRJNA1077598, respectively.
